# Illuminating maternal sepsis: a call for improved recognition and prevention

**DOI:** 10.1172/JCI183887

**Published:** 2024-07-15

**Authors:** Catherine M. Bridges

**Affiliations:** 1 Medical Scientist Training Program, Medical University of South Carolina, Charleston, South Carolina, USA.; 2 Lexington Medical Center, West Columbia, South Carolina, USA.

Late in the afternoon, the intern on the gynecology team texted me, “Come to the OR.” As the sub-intern medical student, I immediately went to find the team. When I entered the OR, I realized they had just finished the emergency uterine evacuation procedure for a patient. I donned gloves to help move her to the recovery area. I noticed her skin was hot. The intern confirmed, “She’s a very sick lady.” She had been waiting for a miscarriage to resolve on its own. Unfortunately, she developed an infection, fever, and, subsequently, maternal sepsis, a systemic immune response to an infection that can quickly turn deadly.

After the procedure, the patient stayed in the hospital for further antibiotics and fluids. I connected with her because we were close in age, and she hoped to bear children, like me. I wondered how a healthy person could become so ill so quickly. In the morning, I was relieved to see our patient was physically healing. She went home the next day. My question of the *how* continued; *maybe she was just unlucky*, I thought. I did not yet ponder the why.

Several months later, my husband and I were overjoyed that I was pregnant. Later, I should have been thirteen weeks along, but the worsening bleeding told me something was drastically wrong. On ultrasound, our embryo had a heartbeat at eight weeks of gestation that was now gone, and our hopes for our first child fled with it. The miscarriage was completed surgically. Days later, shivering, I turned off the AC in my car amid the sweltering summer heat. I knew what to look for. I checked my temperature and watched it soar to over 102 degrees within two hours. My heart raced. I realized, *now, I’m a very sick lady*. I went to the emergency department, and the doctors recognized my maternal sepsis. After a flurry of an emergency procedure, antibiotic treatment, and a two-day hospital stay, I was a survivor. However, the emotional scars remain. I lived the how, so then I asked, *why*?

Recently, I woke up to a text message from my friend containing a news article about Krystal Anderson, a former Kansas City Chiefs cheerleader who died of septic shock after delivering her stillborn daughter (1). *She’s too much like me*, I thought, so I swiped away her message and waited to settle down. *I need to know her*, so I read her story. Like me, Krystal Anderson was African American (1). CDC statistics suggest that “Black women are three times more likely to die from a pregnancy-related cause than White women” (2). *Could her death have been prevented*? Death from maternal sepsis is largely preventable, and women deserve appropriate care (2). *Were we all the unlucky ones*? *Why*?

In their 2017 Statement on Maternal Sepsis, the World Health Organization (WHO) states, “...the third most common direct cause of maternal mortality, maternal sepsis, received less attention, research, and programming” compared with other factors (3). As such, the WHO participated in the Global Maternal and Neonatal Sepsis Initiative to improve outcomes (3). In fact, maternal sepsis is the cause of twenty-three percent of maternal mortality in the United States (4). I was stunned.

I now focus on what can be done about maternal sepsis. In a 2023 review of guidelines for maternal sepsis, Stephens et al. noted that the tools and criteria for diagnosing maternal sepsis were “varied” (5). However, guidelines were “similar in evaluation and management recommendations” (5). This suggests differing guidelines exist to diagnose maternal sepsis even when management is similar. Therefore, an unmet need in maternal sepsis is recognizing the condition. Since the WHO Statement, the guidelines for recognizing maternal sepsis have not been standardized (5, 6). Further biomedical research is needed to standardize diagnostic criteria for maternal sepsis so that healthcare providers can recognize and manage it promptly to save lives.

After focusing on maternal sepsis diagnosis, I was compelled to investigate how it can be prevented before it starts. There are known contributory risk factors for maternal sepsis, from individual to societal, in racial disparities and lack of access to healthcare (6). In my opinion, delayed treatment has recently been illuminated. There are ways to decrease the risk of maternal sepsis (6). Even so, women are still dying unnecessarily. A closer investigation is still needed, especially in diagnosis and prevention.

While I write to bring attention to the insufficient research on maternal sepsis, I further write to bring attention to the increased risks that African American women carry during and after pregnancy. There is a new debate about the exact rate of maternal mortality in the United States (7). However, the racial disparities remain (7). We need a scientific explanation beyond the burdens of racism. I carry maternal sepsis in my life story and wanted to bring attention to my sisters in the stories we share.

## Version 1 07/15/2024

Electronic publication
